# Spatial-Temporal Dynamics of High-Resolution Animal Networks: What Can We Learn from Domestic Animals?

**DOI:** 10.1371/journal.pone.0129253

**Published:** 2015-06-24

**Authors:** Shi Chen, Amiyaal Ilany, Brad J. White, Michael W. Sanderson, Cristina Lanzas

**Affiliations:** 1 Department of Population Health and Pathobiology, North Carolina State University, Raleigh, NC, 27607, United States of America; 2 Department of Biology, University of Pennsylvania, Philadelphia, PA, 19104, United States of America; 3 College of Veterinary Medicine, Kansas State University, Manhattan, KS, 66506, United States of America; University of Namur, BELGIUM

## Abstract

Animal social network is the key to understand many ecological and epidemiological processes. We used real-time location system (RTLS) to accurately track cattle position, analyze their proximity networks, and tested the hypothesis of temporal stationarity and spatial homogeneity in these networks during different daily time periods and in different areas of the pen. The network structure was analyzed using global network characteristics (network density), subgroup clustering (modularity), triadic property (transitivity), and dyadic interactions (correlation coefficient from a quadratic assignment procedure) at hourly level. We demonstrated substantial spatial-temporal heterogeneity in these networks and potential link between indirect animal-environment contact and direct animal-animal contact. But such heterogeneity diminished if data were collected at lower spatial (aggregated at entire pen level) or temporal (aggregated at daily level) resolution. The network structure (described by the characteristics such as density, modularity, transitivity, etc.) also changed substantially at different time and locations. There were certain time (feeding) and location (hay) that the proximity network structures were more consistent based on the dyadic interaction analysis. These results reveal new insights for animal network structure and spatial-temporal dynamics, provide more accurate descriptions of animal social networks, and allow more accurate modeling of multiple (both direct and indirect) disease transmission pathways.

## Introduction

Analysis of animal networks is essential to predict animal behavior, characterize sociality, and investigate many ecological and evolutionary processes, including disease transmission [1]. Many aspects of animal social networks change through time, from global network structure to basic dyadic (between two individuals) interactions [2, 3]. There are two basic types of temporal dynamics on the network: the network structure (intrinsic changes in the network structure), and flow dynamics on the network (e.g. disease transmission, substance and energy flow in food webs [3]). These two types of temporal dynamics, however, are not mutually exclusive as flow dynamics are also closely dependent on network structure changes, and vice versa. A static description of the network may not be able to accurately capture the comprehensive network structure and flows [4, 5].

Recent studies have progressed in developing both concepts and techniques for temporal network dynamics. Temporal network dynamics can take place at different scales: at entire network/global level (how the global network characteristics, such as network density, varies over time), at sub-graph level (how clusters of individuals form and change), at the triadic level (how the transitivity in the triad changes among three vertices), and at the dyadic level (how the relationship of each pair of individuals in the network evolves). Meanwhile, novel computational tools have also been developed to detect and quantify network characteristics and temporal network dynamics, such as quadratic assignment procedure (QAP [6]), exponential random graph model [7–9], modularity detection [10, 11], transitivity test [12], and network comparison across space and time [13, 14].

Temporal networks have been applied to various wild animal populations, from primates to insects, including cyclic population structure in female African baboons [15], social niche stability in primates [16], dynamics of social networks among Asian elephants [17], social system restructure and dynamics in dolphins [18, 19], social network and genetic structure in spotted hyenas [20], social cohesion of marmots remaining at home [21], social network evolution and kin cluster in manakin birds [22, 23], dynamic social networks in guppy fish [24, 25], temporal and individual variation in social ant colonies [26, 27], and triad dynamics in the rock hyrax [28].

A major problem for studies in wildlife ecology is that most of the wild animals are difficult to track individually and continuously with a high sampling rate (resolution) hence the resulting networks are at low temporal resolution, usually at daily level or longer. Furthermore, spatial heterogeneity is also important in animal social networks but often untraceable or ignored [3]. These difficulties substantially hinder the utilization of temporal networks in ecology. These problems can be overcome in studies of networks of domestic animals. Cattle, as many other ungulates, present social interactions such as exploratory behavior, recognition, communication (especially tactile communication), and peer bonding [29]. Although quantitative descriptions of cattle social networks are still uncommon, dynamic social networks have been recorded in both dairy and beef cattle populations [30–32].

Cattle are usually housed in a confined area so that their position can be explicitly and accurately tracked by real-time location system (RTLS) in order to construct ultra-high resolution networks (at second and centimeter resolution temporally and spatially), thus the complete network can be accurately described. Spatial heterogeneity also plays an important role in network dynamics [33, 34], and data at high-resolution for cattle social networks can facilitate better understanding of the complicated spatial-temporal dynamics [32]. Furthermore, the group size of a cattle population is generally constant (excluding immigration and emigration, as opposed to wild animal populations), thus allowing for some computational analysis (e.g. QAP which usually requires the same set of individuals/vertices in the networks). Thus cattle are suitable candidates to study spatial-temporal dynamics of animal networks that can provide insight on the dynamics of such networks in other domestic and non-domestic species.

We here construct high-resolution cattle proximity networks accounting for both temporal (at hourly level in a day) and spatial (different areas in the pen, e.g. feeding bunk, water supply, and hay bunk) heterogeneities. We comprehensively and quantitatively compare and contrast how the network structure changes at different network topology levels, from global network characteristics, to subgraph, triadic, and down to the dyadic interactions, at different time and area, and demonstrate the spatial-temporal heterogeneity at these different levels in a cattle network. This enhances our understanding of animal network dynamics, develops more realistic description and characterization of social networks, and facilitates future research such as incorporating accurate disease transmission models on social networks.

## Materials and Methods

### Data acquisition and standardization

Real-time animal position data were collected at the research farm of Kansas State University (Manhattan, KS, USA). Three pens of Holstein calves were monitored, with 21, 21, and 27 calves in pens #1 to #3, respectively. The entire pen is partitioned into four exclusive areas: around the grain bunk (5m^2^), the hay bunk (5m^2^), the water trough (3m^2^), and all the other areas in the pen (187m^2^). Each calf was continuously monitored from August 11, 2011, to August 18, 2011, using a wide-band radio frequency tag (Ubisense Series 7000 Compact Tag, Ubisense Group, UK) attached to its ear. The tag transmitted ultra wide-band signals to seven receivers around the pens; the receivers then transferred data to a central computer that logs the 2-dimensional (X-Y coordinates) position data. This real-time location system (RTLS) was accurate (up to 0.01 m in spatial resolution and 1s in temporal resolution) and did not disturb the animals nor change their normal behavior. The original position data reported by the tags of each animal were aggregated to 10-s resolution. Hence, in each day, each animal would have 8,640 2-dimensional data points recording its location (24 h/d×60 min/h×6 data/min). The detailed data acquisition and standardization processes are presented in our previous work with indirect and direct contact independently [31, 32]. All the animal experiments associated with this manuscript were approved by and complied with the animal regulation policy of the Kansas State University.

### Network construction

First, the cattle network was generated based on the cattle position data in every hour (a total of 192 hours in this study, thus 192 hourly networks) using a 0.3m threshold distance, as described in our previous work [32] (this work did not take specific spatial areas, e.g. grain, hay, and water into consideration). We use spatial proximity to approximate social closeness in this study (thus proximity network is an approximate to the real social network) because the animals are in a confined area. Then, whenever a direct contact occurs, the positions of the pair are determined and such direct contact is attributed to one of the four areas, using the same 0.3m threshold distance. For example, if two calves are in contact and their position falls within the 0.3m threshold distance of the grain bunk, this direct contact occurs around the grain bunk. Occasionally, a direct contact can be attributed to more than one area: consider a pair of cattle in contact with animal *i* within threshold distance to the grain bunk but animal *j* at the general pen floor. In this situation, priority is given to identify the contact as being associated with the more specific areas (e.g. grain, hay, and water) than the general pen floor, and this contact is assumed to occur at the more specific area. Note that grain, hay, and water themselves in the study pen are sufficiently spatially separated apart that no direct contacts can occur simultaneously in two of them.

The spatially-complete network was partitioned to four spatially-explicit networks representing four different areas in the pen in each hour. These networks are weighted networks (i.e. the values in the adjacency matrix *M* are not binary but assigned numerical values for the number of contacts). For example, if the value of *M*_*ij*_ is *m*, it indicates that two cattle *i* and *j* have a total of *m* contacts within that hour. In this study we assume an undirected network such that *M*_*ij*_ = *M*_*ji*_ (animal *i* contacting animal *j* implies animal *j* contacting animal *i*), such that *M* is a symmetric matrix. The indirect contact structure (i.e. number of contacts between an individual animal and specific areas such as the grain, hay, and water) is also obtained on an hourly basis using the same 0.3m threshold distance, and the time series of indirect contact structure is calculated for these specific areas.

As shown later in the results section, the regular feeding activity (twice a day around 8–9am and 2–3pm, shortened as 8am and 2pm hereafter; food is supplied at the grain bunk) significantly increases cattle contacts during these periods. Our observations also show cattle usually gather around hay to ruminate shortly after feeding. Thus in each day the feeding time (8am and 2pm) is separated from all other non-feeding time. We first investigate the potential coupling and synchronization between indirect contact (with grain, water, and hay) and direct contact using time series analysis and spectrum analysis. The time series (at hourly level) between indirect and direct contact are plotted for the entire 192-h period. The coherency between indirect and direct contact is computed and plotted to quantify such synchronization. If the mean frequency coherency is above certain threshold (sampling frequency) then there is significant correlation between the two (direct and indirect contact) time series. This step is necessary to reveal temporal heterogeneity in network structure and its potential cause. Then we characterize and compare the network structure dynamics at different times of day and at different areas in the pen to delineate the spatial-temporal heterogeneities in the network.

### Network characterization and comparison

A range of properties and statistics are used to characterize the hourly network topological structure, from a global level down to dyadic interactions. The network density is one of the most important and common measure of networks at a global level. In our study system (weighted network), the network density is proportional to the total number of contacts in each hour. The network density is bracketed between 0 and 1, where 0 indicates no contact in that time period (1-h) at all and 1 representing a fully connected network (i.e. each pair of vertices is connected).

Furthermore, the vertices in the network (in our study system, the cattle) can show a clustering pattern and form groups. Modularity measures the strength of the division into clusters/groups. Modularity can be positive or negative, and a larger positive value indicates stronger clustering pattern [35]. At the triadic level (three vertices), transitivity, also known as the global clustering coefficient, measures to what extent vertices tend to cluster together [12]. The means and standard errors of modularity and transitivity are also obtained from these networks.

In order to compare network change over time and across space at the dyadic level (two vertices, i.e. whether two cattle consistently act together), the QAP is applied. The QAP correlation is an extension of common Pearson’s correlation, and measures how dyadic interaction changes through time for different networks with the same set of vertices. A larger QAP correlation indicates more consistent and stable dyadic interaction between two networks at different times. As indicated before, the time (hour) of day is considered either “feeding” (*F*) or “non-feeding” (*N*). For all the 192 observed networks for each area, there are a total of 16 networks during feeding time (8am/2pm, for 8 days) and 176 for non-feeding time. The QAP is applied between each pair of any two networks during feeding time (a total of 16*15/2 = 120 pairs *FF*), between each pair of networks during non-feeding time (a total of 15,400 pairs *NN*), and between each pair of networks in feeding and non-feeding times (a total of 1408 pairs *FN*). All these QAP correlation values are compared between different time periods of the day as well as for different areas in order to reveal dyadic interaction changes. All the analyses were performed in *R* 3.1.0, with the *statnet* package (version 1.90).

## Results

**Fig 1:**
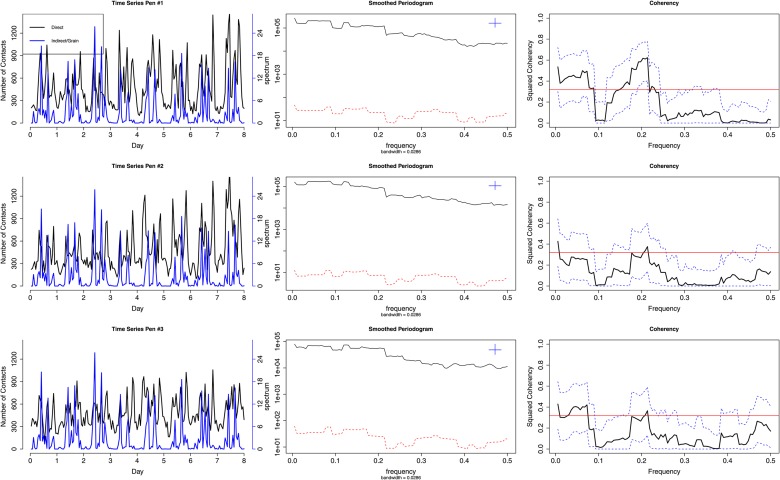
Time Series of Indirect Contact (Grain, Water, Hay), Periodogram, and Coherency Plot. Note: The subfigures are for grain (upper), water (middle), and hay (lower), respectively. In these subfigures, the time series plots (left panels) of indirect contact and direct contact show high synchrony (coupling) in grain and hay, as supported by the coherency plots (right panels, solid black line) that have substantial part above the threshold (solid red line). There is no clear pattern of relationship between indirect contact with water and direct contact.

**Fig 2:**
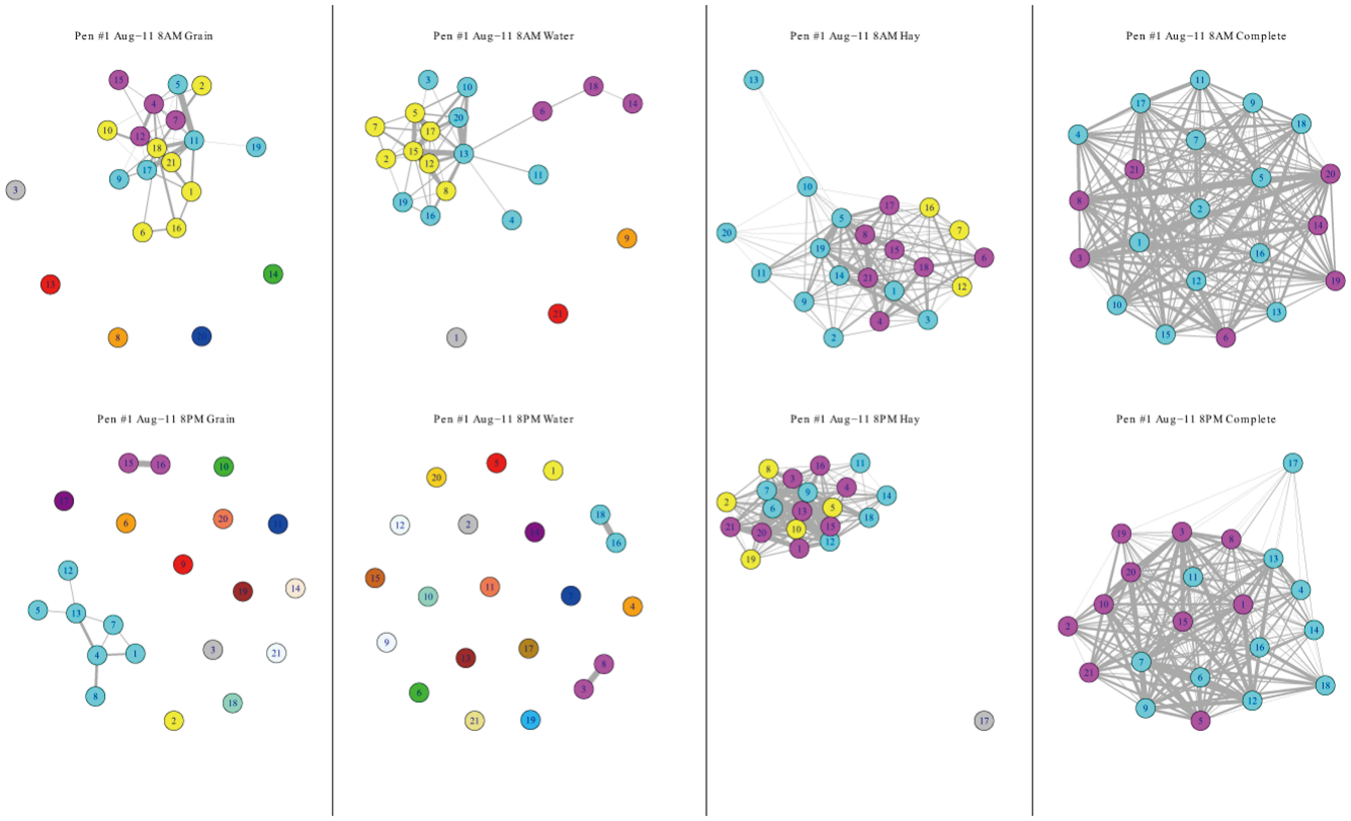
Network Structure with Potential Clustering Examples at Different Time and Different Area. Note: from top to bottom: networks around grain, hay, water, and complete network in the pen. Left: networks aggregated during 8–9AM on Aug. 11. Right: networks aggregated during 8–9PM on Aug. 11. Different colors indicate potential clustering (subgraph) based on the analysis in *R* with *statnet* package. The thickest line in each network corresponds to the largest number of contacts for that particular area in one hour, thus not directly comparable between different networks. Networks in grain, hay, and water show strong spatial-temporal heterogeneity and distinctive clustering pattern, but such heterogeneity diminishes when aggregated to the complete networks.

The time series of calf network density (proportional to number of contacts, at hourly level) for the complete observation period (192-h in total) is plotted against the time series of indirect contact between cattle and grain, water, and hay for Pen #1 as an example (Fig 1). The time series of direct contact show substantial diurnal cycle, and the time series between direct and indirect contacts at grain and hay (upper and lower panels, respectively) are significantly coupled, which is supported by the coherency plot in the right panels of Fig 1 (potential synchronization occurs since the solid black line is above the red significance level). For instance, the coherency plot for direct contact and indirect contact with grain shows a peak (must be above the red significance level) at around 0.2, which corresponds to an approximately 5-h synchronization between direct contact and indirect contact with grain. However the time series between direct and indirect contact with water (middle panel) are not significantly correlated (the solid black mean coherency line does not exceed the significance level, the red horizontal line). Pen #2 and pen #3 have similar results as pen #1 that direct contact is significantly correlated to indirect contacts with grain and hay, but not with water (though not shown in the figure). These results show that cattle network density changes significantly within a day, and feeding activity promotes clustering (thus more dense networks) around grain and hay, while drinking is not likely a key factor for network changes. These results indicate that there is neither temporal stationarity nor spatial homogeneity in this high-resolution cattle network (number of contacts). Explicit examples of the real cattle networks with possible modules (clustering) at different time and location are provided in Fig 2, illustrating strong temporal-spatial heterogeneity in network structure without quantitative measurements: the cattle change their aggregation at grain and water in different time periods (8AM vs 8PM) such as number of contacts and potential cluster. However, temporal heterogeneity vanishes if we look at the networks around hay and the spatially-complete network, where the two time periods yield similar structured networks.

**Fig 3:**
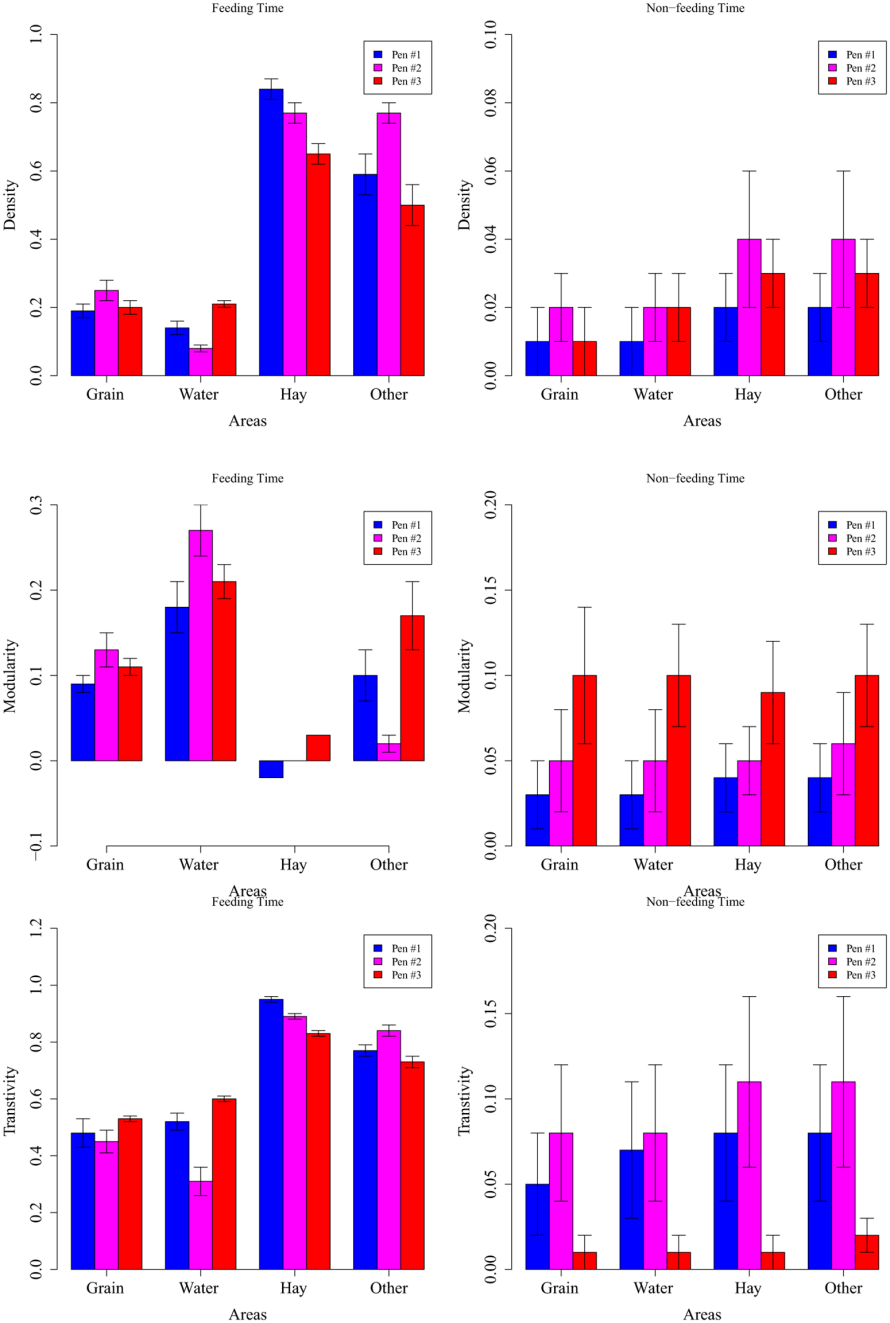
Network Structure Characteristics (density, modularity, and transitivity) at Different Time and Different Area. Note: Subfigures are for network structure characteristics: density, modularity and transitivity values, respectively. Left: feeding time (between 8AM-9AM and 2PM-3PM); right: non-feeding time (other time periods). Note all the network characteristics differ significantly between feeding and non-feeding time (left vs right panel in each sub-figure). Spatial heterogeneity (different areas) does not have significant impact during non-feeding time but substantially alter network characteristics during feeding time.

Next, we show three important network measurements (density, modularity, and transitivity, covering levels from the global network structure to dyadic interactions) in different time periods (feeding time were during 8AM-9AM and 2PM-3PM, shortened as 8AM and 2PM thereafter, and non-feeding time were all the other time periods in a day) and in different areas (grain, water, hay, and other pen floor) to explicitly demonstrate the spatial-temporal heterogeneity (Fig 3). The network density measures at a global network structure level and can be approximated as the number of total contacts. It is not surprising that network density is much higher during feeding time than non-feeding time (Fig 3, first row), as supported by the time series plot in Fig 1. Interestingly, the cattle have more contacts around hay than grain even during feeding time. This can be explained by the fact that the food supply at the grain is limited and feeding behavior usually only lasts for 15–20 minutes according to our observation^31^. However the cattle spend significantly longer time (about 45–60 minutes) at hay after feeding to ruminate, and during rumination calves tend to gather together. For the grouping and clustering of the networks, results reveal very low modularity value (almost 0) around hay during feeding time, which indicates almost all cattle have some connection with others (Fig 3, 3^rd^ row, left panel). This is very distinct from around the grain, water, and hay during feeding time, and from all areas during non-feeding time.

During feeding time, water area has the largest modularity value (0.18–0.27, Fig 3, 3^rd^ row). Although the modularity values indicate cattle have some connection with others at hay during feeding time (-0.02–0.03), it is then revealed by the transitivity values that they actually divide into more stable subgroups at hay during feeding time (0.83–0.95, Fig 3, 4^th^ row, left panel). This paradoxical result can be explained by the fact that during feeding time, the cattle tend to cluster into several subgroups and these subgroups are not isolated around hay (i.e. different subgroups are connected by some “bridge” vertices/individuals). Such a network shows low modularity (i.e. vertices are more or less connected) but high transitivity (i.e. the vertices form distinctive subgroups). The transitivity values are not substantially different among different areas during non-feeding time. Pen #3 has almost the same transitivity values as pen #1 and pen #2 during feeding time, but it yields a smaller transitivity than other pens during non-feeding time. Such pen-level difference can be explained by the fact that transitivity values are generally very low (<0.1) during non-feeding time and the pen-level difference is more identifiable.

**Fig 4:**
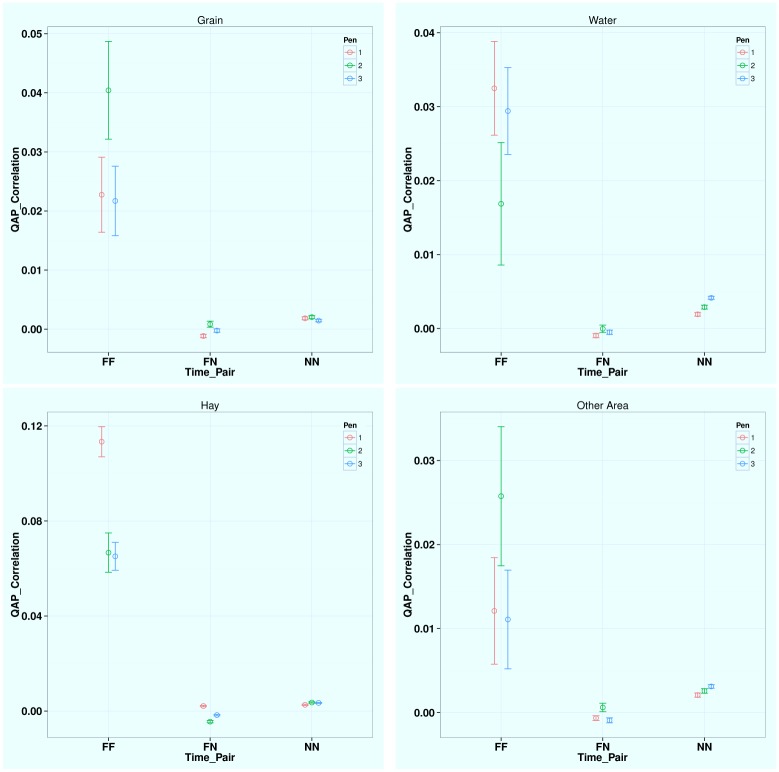
QAP Correlation Test Results for Different Time Period Pairs at Different Areas. Note: the four sub-figures are for grain, water, hay, and all the other areas, respectively. *F* indicates feeding time period (8–9AM, 2–3PM) and *N* indicates non-feeding time periods. Within each subfigure (the same area) QAP is significantly larger between two feeding time periods (*FF*) than between one feeding and one non-feeding time periods (*FN*) and between two non-feeding time periods (*NN*). Hay has the much larger QAP correlation during feeding time (FF, valued at about 0.1) than grain (0.03), water (0.02), and all the other area (0.02). Thus the contact network structure especially dyadic interaction is most consistent and stable during feeding time around hay.

At the dyadic level, the correlations are much stronger (larger quadratic assignment procedure, QAP correlation values) between any two feeding time periods (*FF*) than between any two non-feeding time periods (*NN*) and between one feeding and one non-feeding time periods (*FN*), which indicates more stable and consistent dyadic interactions during feeding time at all four locations (see Fig 4). Thus feeding tends to promote and preserve the network structure. For different areas during feeding time, hay has significantly higher QAP correlation value (0.10) than grain (0.03), water (0.02), and all other pen floors (0.02), meaning cattle tend to choose their partners and interact with others more consistently around hay during feeding time.

## Discussion

Our results clearly reveal that the cattle network has substantial spatial and temporal heterogeneity. The network structure, from global characteristics to basic dyadic interactions, changes during different times of the day as well as in different areas. Most of the contacts (approximately 60% of total contacts) happen during the feeding time (a total of 2 hours per day, which accounts for only 8% of total daily time). Interestingly, hay accounts for more direct contacts during feeding time than grain, as can be seen from the network density plot (Fig 2). It also coincides with our previous finding that cattle spend significantly longer time around hay (about 2 hours per day) than around the grain bunk (about 1 hour per day, 31 Chen et al. 2013). These results show the possible correlation between direct animal-animal contacts and indirect animal-environment contacts. However, the spatial-temporal heterogeneities in the cattle network diminish when the networks are aggregated at lower temporal (e.g. at daily level or above) and spatial (e.g. aggregated at entire pen) resolution. A major challenge to current studies of dynamic networks is lack of sufficiently resolved data, especially for wildlife studies [2, 36–39]. Thus cattle (and other domestic animals) serve as an excellent example to study and understand real-time evolution of network structure and potentially shed light on wildlife studies, especially the optimal observation strategy. For instance, researchers can observe animal social network during specific short period of time while preserving most of the network structure characteristics

Except very specific behavioral interactions, many animal social networks are constructed based on the assumption that spatial proximity reflects social closeness. But this assumption may be incorrect because distance alone (and hence contact) does not necessarily reflect real social ties [3]. Adult cattle have approximately 2m flight distance (an animal will change normal behavior and move if get closer than the flight distance) and young calves have smaller flight distance [29]. When a young calf is less than the threshold distance (0.3m in this study) from other calves, it does not necessarily imply the two calves are having some social interaction—they may be just randomly passing by each other. The results, especially QAP values, from our study show that the dyadic interaction is unstable and inconsistent during non-feeding time periods. Thus, we argue that the some of the contacts during non-feeding time are not real social interactions but perhaps just random contacts. During feeding time, social networks formed around hay are much more stable than at the grain bunk. We suggest that during feeding time, the cattle need to compete with others for food in the grain bunk thus cannot always eat with an intentionally chosen partner. On the other hand, after feeding the cattle can go with their partner freely to the hay for feeding. Thus the contacts around the grain bunk are facilitated by feeding activity but may not necessarily reflect social ties. It is only the contacts around the hay bunk during feeding time that may attribute to the real social ties. Thus based on the spatial and temporal heterogeneity in the social network, we can categorize the contacts into three types: social, random, and indirect-contact facilitated contact. Our study provides a potential way to infer the real social network based on the observed proximity network.

One of the major applications of dynamic networks is to model disease transmission on the animal or human networks. Recent studies have already revealed the importance of spatial-temporal heterogeneity in the contact structure for disease transmission in direct animal-animal contact pathway and indirect animal-environment pathway [31, 32]. Synthetic data are used to simulate multiple transmission pathways simultaneously but the different pathways are considered uncorrelated as well [40]. Our results demonstrate that indirect contact between animal and environment and direct contact between animals can be highly synchronized and coupled (especially during feeding time). This enables future research on modeling multiple transmission pathways at the same time, explicitly investigating the relative importance of each transmission pathway (e.g. direct contact, foodborne, waterborne, etc.), and measuring the effects of spatial-temporal heterogeneity with a realistic high-resolution social network.
